# Attenuation of Immune Senescence Markers After Intensive Cancer Therapy Through Resistance Training: A Pilot Study

**DOI:** 10.3390/cancers18111710

**Published:** 2026-05-24

**Authors:** Laura F. Newell, Eric Twohey, Jason Sweetnam, Sasha Skendzel, John Stingle, Kristina A. Vartanian, Brett A. Davis, Cora E. Layman, Lucia Carbone, Karina Ray, Suzanne S. Fei, Lisa Karstens, Fiona C. He, Najla El Jurdi, Anne H. Blaes, Gabrielle Meyers, Rachel J. Cook, Austin Baraki, Donald R. Dengel, Shernan G. Holtan

**Affiliations:** 1 Knight Cancer Institute, Hematology and Medical Oncology, Oregon Health & Science University, Portland, OR 97239, USA; 2 School of Medicine, University of Minnesota, Minneapolis, MN 55455, USA; 3 Personal Trainer, Minneapolis, MN 55455, USA; 4 School of Nursing, University of Minnesota, Minneapolis, MN 55455, USA; 5 M Health Fairview, Nutrition Services, Minneapolis, MN 55455, USA; 6 Integrated Genomics Laboratory, Oregon Health & Science University, Portland, OR 97239, USA; 7 Department of Medicine, Knight Cardiovascular Institute, Oregon Health & Science University, Portland, OR 97239, USA; 8 Bioinformatics & Biostatistics Core, Oregon National Primate Research Center, Oregon Health and Science University, Beaverton, OR 97239, USA; 9 Biostatistics Shared Resources, Knight Cancer Institute, Oregon Health & Science University, Portland, OR 97239, USA; 10 Division of Oncological Sciences, Knight Cancer Institute, Oregon Health & Science University, Portland, OR 97239, USA; 11 Division of Informatics, Clinical Epidemiology and Translational Data Science, Department of Medicine, Oregon Health & Science University, Portland, OR 97239, USA; 12 Division of Hematology, Oncology, and Transplantation, Department of Medicine, University of Minnesota, Minneapolis, MN 55455, USA; 13 Department of Medicine, Brooke Army Medical Center, Fort Sam, Houston, TX 78234, USA; 14 School of Kinesiology, University of Minnesota, Minneapolis, MN 55455, USA; 15 Roswell Park Comprehensive Cancer Center, Carlton & Elm, Buffalo, NY 14263, USA

**Keywords:** resistance training, cancer survivorship, immunosenescence, hematopoietic cell transplantation, inflammation and immune aging, exercise immunology

## Abstract

**Background:** Chemotherapy and radiation accelerate aging of multiple systems, including the immune and musculoskeletal systems. Resistance training may mitigate some of the late physiologic effects of cancer therapy. **Methods:** We developed a community-based pilot study of resistance training for long-term cancer survivors meeting criteria for pre-frailty or frailty (*N* = 8; 6 allogeneic hematopoietic cell transplant, 1 autologous hematopoietic transplant, 1 breast cancer survivor) and their caregivers (*N* = 8 healthy controls) consisting of a baseline assessment, 10 weeks of personalized resistance training at least once weekly as a group and as many additional times on an individual basis as their schedule allowed, and an end-of-study assessment to measure change in strength and body composition. Blood samples were collected at the start of the study and after the 10-week training program to assess changes in peripheral blood mononuclear cell DNA methylation patterns, gene expression measured by RNA sequencing, and stool microbiome analysis using metagenomics. The median number of resistance training sessions was 25 sessions. **Results:** Cancer survivors and controls both more than doubled their squat and press volume after 10 weeks. At baseline, cancer survivors exhibited a pro-inflammatory transcriptomic and epigenetic profile with elevated interferon signaling and reduced naïve T cell signatures compared to healthy controls, consistent with immune senescence. After 10 weeks of resistance training, these differences normalized, suggesting that exercise exerted anti-inflammatory and immune-restorative effects in cancer survivors at both gene expression and methylation levels. Ten fecal microbial pathways that were lower in relative abundance in patients compared with controls at baseline were no longer significantly different post-exercise. **Conclusions:** Our data suggest that in addition to beneficial changes in body composition, resistance training may exert an immune restorative effect in cancer survivors.

## Simple Summary

Cancer treatments such as chemotherapy and radiation can accelerate aging of the body, leading to weakness, inflammation, and impaired immune function. We conducted a pilot study in cancer survivors and their caregivers to test whether a 10-week resistance training program could improve physical and biological health. Participants significantly increased their strength and showed improvements in body composition. Importantly, cancer survivors started with signs of chronic inflammation and immune aging, but these differences were no longer present after the exercise program. Changes were also seen in DNA methylation and the gut microbiome, suggesting broader biological effects. These findings suggest that resistance training may help restore physical strength and improve immune health in cancer survivors, supporting exercise as an important part of cancer recovery and survivorship care.

## 1. Introduction

Cancer therapy can accelerate aging of multiple physiologic systems, resulting in an increased risk of frailty, a clinical syndrome of diminished physiologic reserve and low resistance to stressors [1,2,3,4]. Frailty can manifest as weakness, low physical activity, exercise intolerance, unintentional weight loss, self-reported exhaustion, and low self-efficacy [5,6]. While its manifestations are common and undoubtedly recognized as expected or “normal” side effects of therapy, frailty is likely underdiagnosed in cancer survivors [7]. Notably, therapy-induced frailty is not limited to older adults; younger cancer survivors, including recipients of hematopoietic cell transplantation, may also develop features of accelerated physiologic aging. Given that malnutrition, fatigue, and weakness symptoms are so common during and after cancer therapy, often with greater focus on treatment-specific side effects and outcomes, the multisystem effects that can culminate in accelerated physiologic aging may not be adequately addressed. Therefore, there is an unmet need to both increase the recognition of the effects of cancer diagnosis and cancer therapy that exert deleterious effects on the hallmarks of aging, and to develop a multidisciplinary approach to prevent or mitigate these effects.

Resistance training programs can improve skeletal muscle mass, as well as cardiovascular health, insulin sensitivity, and all-cause mortality, and directly address multiple domains of frailty [8,9]; such training programs have shown success in other clinical settings, including community-dwelling elderly patients [10], those residing in long-term care facilities [11], and after lung transplantation [12]. Level 1 evidence to support exercise in oncology now exists, with a randomized trial showing a disease-free survival benefit in the adjuvant colon cancer setting [13]. The mechanisms supporting the survival improvement results are not fully known. We thus performed a community-based pilot study of strength training in long-term survivors of cancer, with a particular focus on allogeneic hematopoietic cell transplantation (HCT). The results obtained from this cohort offer valuable physiologic and preliminary genetic/epigenetic insights into the potential benefits of resistance training for cancer survivors. These preliminary data are useful to inform the development of a larger strength-focused longitudinal training program to reverse frailty and improve all possible domains of physiologic and social function after cancer therapy, with a particular emphasis on resolution of inflammation and reversal of immunosenescence.

## 2. Patients and Methods

### 2.1. Strength Training Pilot Study

We designed a pilot study of community-based strength training specifically to address frailty. Participants (*N* = 8) were required to be at least 18 months post-HCT or -intensive multi-agent chemotherapy, meet criteria for pre-frailty or frailty per Fried et al. [5] (all had low energy expenditure and self-reported exhaustion), and have a healthy exercise partner (caregiver, spouse, other relative, or friend) who could serve as a control (*N* = 8). Among cancer survivors, six patients had undergone allogeneic HCT, one autologous HCT for lymphoma, and one intensive chemotherapy for breast cancer. At the time of the study, all subjects were at least 18 months post-treatment and were receiving <20 mg of prednisone/equivalent. The study was originally intended for allogeneic HCT recipients, but given preliminary benefits with the first cohort of 4 patients, we expanded eligibility to anyone who had received intensive chemotherapy. Two patient cohorts were enrolled between 2018 and 2019. The study was closed to new enrollment early given the challenges the COVID-19 pandemic posed to safe gym access for immunocompromised individuals.

Participants underwent an initial assessment by a National Academy of Sports Medicine-certified trainer, who developed an individualized strength training program. Over the subsequent 10 weeks, participants completed supervised group sessions at least once weekly and were encouraged to perform additional unsupervised sessions following the same protocol, which were self-reported. Each program included three lower-body, four upper-body, and three core exercises, along with one aerobic test. Exercise intensity was prescribed based on repetitions required to approach muscular failure at baseline. Most exercises were performed on Freemotion^®^ machines (Freemotion Fitness, Logan, UT, USA), which use a rotating cable system to accommodate varying body types and allow a natural range of motion.

During the study, specific exercises and their loads were progressed according to each participant’s abilities. All the participants’ workouts (both those with and without a trainer) were recorded and collected, and the results were calculated by comparing subjects’ initial assessments with their final workouts. To assess strength adaptation after 10 weeks, we determined training volume (repetitions completed in a set multiplied by weight lifted) for each of the muscle groups, comparing the initial and final sessions using descriptive statistics and non-parametric Mann–Whitney tests. All participants were given a Fitbit^®^ (Google Fitbit, San Francisco, CA, USA) at the start of the study and encouraged to wear it as much as possible over the course of the study to allow tracking of activity and sleep. Fitbit^®^ data were downloaded from Fitabase^®^ (Small Step Labs, LLC, San Diego, CA, USA) for statistical analysis.

### 2.2. Body Composition and Nutrition

Body composition (weight, body fat percentage, and muscle mass) was measured at baseline and study completion using an InBody^®^ (InBody Co., Ltd., Seoul, Republic of Korea) bioelectrical impedance device. Participants were advised that the goal was muscle gain rather than weight loss. No formal dietary intervention was implemented; however, a registered dietitian (JS) provided monthly group sessions focused on nutrition to support muscle growth.

### 2.3. Genomics Analyses

Of the total cohort, cancer survivors (*N* = 5) and their matched healthy controls (*N* = 5) who had the most exercise sessions during the study were selected for analysis of cryopreserved peripheral blood mononuclear cell (PBMC) methylome and transcriptome profiles.

### 2.4. RNA Sequencing

RNA sequencing was performed on 20 samples (5 cancer survivors pre- and post-training and 5 healthy controls pre- and post-training). In brief, total RNA was prepared from approximately 2–4 million peripheral blood mononuclear cells per sample, using the RNeasy Mini kit and including DNAse-treatment (Qiagen, Valencia, CA, USA). RNA was profiled using a 2100 Bioanalyzer (Agilent Technologies, Santa Clara, CA, USA) to get RNA Integrity Numbers (RIN). RNA evaluated as sufficiently intact was then processed using the TruSeq Stranded mRNA protocol (Illumina) with 200 ng of total RNA input. Briefly, poly(A)+ RNA was isolated using oligo-dT coated magnetic beads. The recovered RNA was then fragmented using heat and divalent cations. The fragment RNA was converted to double-stranded cDNA using random hexamer primers. The second strand was synthesized using dTTP to enforce stranded library construction. The cDNAs were adenylated, then ligated to an Illumina sequencing adapter. The final library was amplified by limited rounds of polymerase chain reaction and cleaned using AMPure beads (Beckman Coulter, Brea, CA, USA). The libraries were profiled on a TapeStation 4200 (Agilent Technologies, Santa Clara, CA, USA), quantified by real-time PCR using an NGS library quantification kit (KAPA/Roche, Basel, Switzerland) on a QuantStudio 3 Real-Time PCR workstation (Applied Biosystems, Thermo Fischer Scientific, Waltham, MA, USA), and sequenced on a NovaSeq 6000 (Illumina, San Diego, CA, USA).

Raw sequencing reads were quality-checked using FastQC v0.12.1, and summary reports were aggregated using MultiQC v1.35. Adapter trimming and quality filtering were conducted with Trimmomatic v0.39. Reads were trimmed using a 4-base sliding window approach, removing reads when the average quality within the window fell below a Phred score of 15. Reads shorter than 36 base pairs after trimming were discarded. On average, less than 0.5% of reads were removed per sample, indicating high initial sequencing quality.

Trimmed reads were aligned to the human reference genome (GRCh38.103, Ensembl) using the STAR aligner v2.7.11b. STAR performs fast and accurate alignment through a two-pass mapping strategy against an indexed genome. Alignment rates for all samples were consistently around 90%, indicating efficient and reliable mapping.

Gene-level expression quantification and normalization were carried out using the EdgeR v4.8.0, DESeq2 v1.48.1, and Limma v3.62.2 packages in R [14,15,16]. Genes were retained for analysis if they were expressed above background levels in most samples. Differential gene expression analysis was performed to compare expression levels between groups. Genes were considered differentially expressed if the corresponding statistical test yielded an adjusted *p*-value < 0.05 (Benjamini–Hochberg correction) and an absolute log2 fold-change ≥ 1, corresponding to at least a two-fold difference in expression.

### 2.5. DNA Methylation

DNA was prepared from approximately 2–4 million peripheral blood mononuclear cells, using the Qiagen QIAamp DNA Blood Mini kit. DNA methylation was assessed in 20 samples using Reduced Representation Bisulfite Sequencing (RRBS). Briefly, 100 ng of genomic DNA per sample was digested with *MspI* (New England Biolabs, Ipswich, MA, USA), which cleaves at CCGG sites to enrich for CpG-rich regions. Library preparation was performed using the NEBNext Ultra II DNA Library Prep Modules with NEBNext Methylated Adaptors (New England Biolabs). Ligated DNA fragments were size-selected using Ampure XP magnetic beads (Beckman Coulter) to generate libraries with an average fragment size of approximately 200 bp.

Bisulfite conversion was conducted using the EZ-96 DNA Methylation-Gold Kit (Zymo Research). Libraries were PCR-amplified using NEBNext Q5U polymerase and barcoded with NEBNext Multiplex Oligos for Illumina (New England Biolabs). Final libraries were purified using Ampure XP beads, normalized, and pooled for paired-end sequencing on the Illumina NextSeq 500 platform.

Raw reads were quality-trimmed using TrimGalore with a Phred score threshold of 20. Trimmed reads were aligned to the bisulfite-converted human reference genome (GRCh38.103, Ensembl) using Bismark, which performs in silico bisulfite conversion of both reads and reference prior to alignment. Methylation status at each CpG site was inferred based on the presence of cytosine (methylated) or thymine (unmethylated). Alignment rates ranged from 73% to 78%, consistent with high-quality bisulfite sequencing. Non-CpG methylation rates were below 1%, indicating efficient bisulfite conversion.

Differentially methylated regions (DMRs) were identified using the methylKit package. Only CpG sites with a minimum coverage of 5× and below the 99.9th percentile of coverage were included. The genome was tiled into 1000 bp non-overlapping windows, and average methylation rates were computed per tile. Tiles were required to contain at least 8 covered CpGs and be represented in at least 7 samples per group. For each tile, a methylation difference (treatment minus control) and q-value (adjusted *p*-value) were calculated. DMRs were defined as regions with a q-value < 0.1 and an absolute methylation difference > 10%.

Hypothesis-generative pathway enrichment analyses were performed on DMRs and differentially expressed genes (DEGs) using Enrichr^®^ [17]. Specifically, MySigDB 2020, WikiPathways 2024 Human, CellMarker 2024, and Reactome Pathways 2024 databases were queried.

### 2.6. Microbiome Analysis

Raw sequencing data were processed using the KneadData v0.12.0 pipeline, incorporating Trimmomatic for quality control and Bowtie2 for host read removal. Microbial taxonomic and functional profiling was performed using the bioBakery meta-omics suite [18], with MetaPhlAn4 v4.0.6 for taxonomic classification (CHOCOPhlAn database) and HUMAnN3 v3.0.1 for gene family and pathway quantification [19]. Outputs were normalized using the humann2_renorm_table script with default HUMAnN3 settings. Downstream analyses were conducted in R 4.4.1. Alpha diversity was assessed by the Shannon index (vegan v2.7-1), while beta diversity was evaluated by Bray–Curtis principal coordinates analysis. Multivariable associations were tested using MaAsLin3 v0.99.16 [20], and group-level differences were assessed by PERMANOVA using the adonis function in vegan.

### 2.7. Ethical Considerations

The University of Minnesota Institutional Review Board approved the resistance training pilot study. The clinical trial is registered on ClinicalTrials.gov as NCT03609203.

## 3. Results

### 3.1. Strength Training Pilot Study Cohort

Eight adult patients who had undergone intensive chemotherapy and eight healthy controls completed the strength training pilot study, consisting of a baseline assessment, 10 weeks of personalized and supervised strength programming at least once weekly as a group, and an end-of-study assessment to measure progress. Demographics of the enrolled participants are in Table 1.

### 3.2. Strength Training Pilot Study Outcomes

There were no serious adverse events related to strength training. The median number of strength training sessions completed over 10 weeks by participant was 25 (range 6–34, Supplementary Data S1). Changes in biometrics are summarized in Supplementary Data S1. Although this was not designed as a weight-loss study, most participants lost weight while gaining lean mass. Patients lost on average 2.45 pounds, while controls lost 6.15 pounds (*p* = 0.56). Both patients and controls showed decreases in body fat percentage and fat mass, with reductions of up to 15.6 pounds and 20 pounds in one patient and one control, respectively. The ratio of extracellular water to total body water (ECW/TBW), an indirect measure of inflammation, decreased over time in study participants, although this was not statistically significantly different between patients and controls.

Changes in training volume by exercise type are detailed in Supplementary Figure S2A–G. Study participants increased their exercise volume across all exercise types, with no statistically significant differences between patients and controls in the percent increase in volume over the 10-week training period. Both patients and controls could more than double their training volume (increases of ~150% across groups; Figure S2A,E) for the free-motion squat and shoulder press movements.

### 3.3. Fitbit Analysis

There was no statistically significant difference in Fitbit accelerometer data between cancer survivors and controls (Supplementary Data S1). The median Fitbit wear time per day was 19.1 h (range 5.6–22.5 h). The median duration of sleep per night was 6.7 h (range 1.4–6.8 h), with REM sleep having a median of 1.4 h (range 0–1.6 h). The median daily resting heart rate was 66.2 beats per minute (BPM, range 63.1–82 BPM), and the median daily maximum heart rate was 122.1 BPM (range 112.5–157.6 BPM). The median number of steps per day was 5839 (range 3387–8825 steps). The median number of active/very active minutes per day was 16.4 min (range 2.1–35.8 min). The median estimated number of calories burned per day was 2334 calories (range 1596–3151 calories).

### 3.4. RNA Sequencing

#### 3.4.1. Pre-Resistance Training Assessment

**Figure 1:**
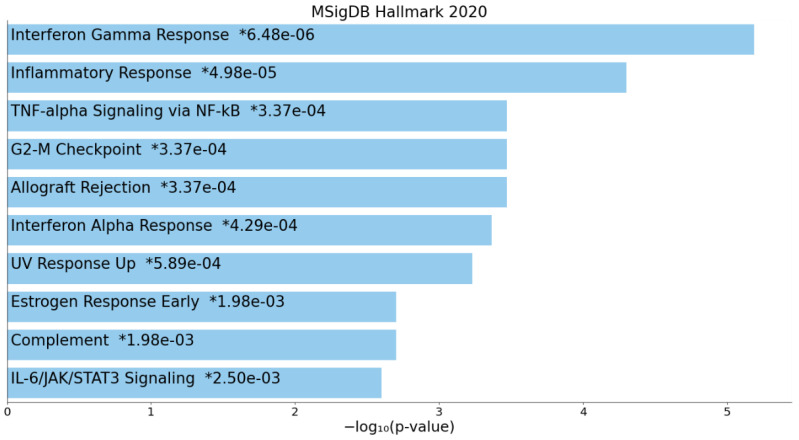
Top 10 enriched hallmark pathways in cancer survivors compared to healthy controls prior to initiation of resistance training (MySigDB 2020).

Overall, there were 130 upregulated and 625 downregulated DEGs in cancer survivors compared to healthy controls before resistance training (i.e., baseline) (Supplementary Data S2). Specifically, cancer survivors’ PBMC gene expression profiling showed marked expression of inflammation-related genes compared to healthy controls (Figure 1). The most significantly enriched pathway was the interferon gamma response, indicating a dominant signature of type II interferon signaling. This was followed by enrichment of inflammatory response, TNF-alpha signaling via NF-kB, and interferon alpha response.

Simultaneously, analysis of downregulated genes revealed a relative deficiency in naïve T cell generation in cancer survivors compared to controls at baseline (Table 2). This finding, coupled with the activated inflammatory state marked by interferon signaling and possible T cell effector differentiation or exhaustion, could be consistent with immune senescence.

#### 3.4.2. Post-Resistance Training Assessment

The resistance training intervention resulted in 470 upregulated and 813 downregulated DEGs in cancer survivors. Cancer survivor gene expression before resistance training, compared with their post-training profiles, suggested a baseline dysfunctional metabolic state (Table 3). The most enriched pathway from pre- to post-exercise was muscle catabolism; NAD, sirtuins and aging were also present in the top enriched pathways [21].

There were no statistically significant differences in gene expression in cancer survivors compared to controls post-exercise, suggesting normalization of the differences in immune-related genes and pathways present before exercise. The transcriptome of healthy controls also did not significantly change pre/post-exercise (i.e., 0 DEGs). This suggests that, despite the anthropometric improvements observed with resistance training in both cohorts, cancer survivors had a greater potential immunologic benefit from resistance training after 10 weeks than healthy controls.

**Figure 2:**
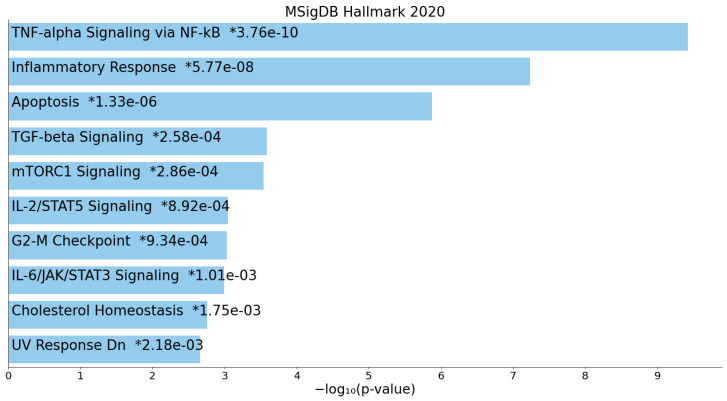
Top 10 enriched hallmark pathways in cancer survivors prior to initiation of resistance training compared to post-exercise (MySigDB 2020).

Core inflammatory pathways were also elevated at baseline in cancer survivors and were attenuated by resistance training (Figure 2). Prior to exercise, elevated TNF-alpha signaling, inflammatory response, IL-6/JAK/STAT3, and TGF-beta pathways suggested chronic low-grade inflammation or immune dysregulation at baseline [22,23,24]. The lack of differences in these pathways after training suggests the hypothesis that resistance exercise could potentially exert a normalizing effect.

### 3.5. DNA Methylation

Overall, there were more DMRs in cancer survivors than in controls at study start (1479 DMRs) than at study completion (924 DMRs) (Supplementary Data). Moreover, resistance training appeared to cause greater epigenetic changes in cancer survivors. Resistance training was associated with changes in DMRs across 559 genes in cancer survivors, compared with only 109 genes in healthy controls. Enrichr^®^ pathway analysis showed that genes involved in the innate immune response, particularly Toll-like receptor genes, were less methylated in cancer survivors after exercise (Table 4).

While there were no statistically significant differences in DMRs between cancer survivors and controls pre- or post-exercise, the analysis of DMRs and DEGs revealed a gene set consistent with substantial differences in naïve T cells before exercise (Table 5). This significant difference between cancer survivors and controls was no longer present after resistance training (i.e., 0 DMRs).

### 3.6. Microbiome

**Figure 3:**
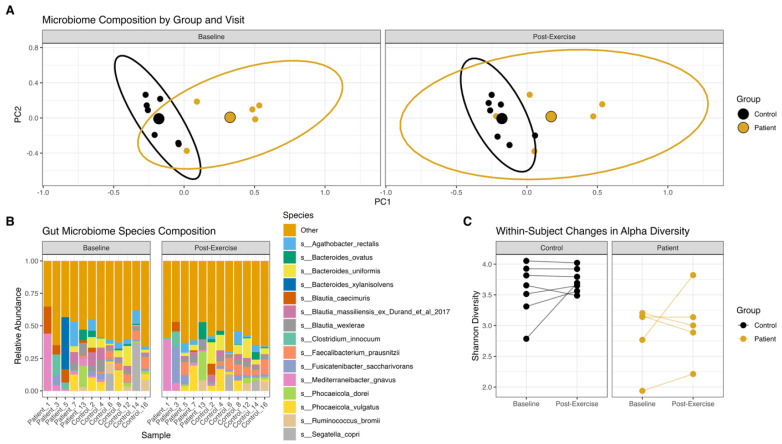
Gut microbiome composition and diversity. (**A**) Principal coordinates analysis (PCoA) of microbial community composition. Points represent individual samples, and ellipses indicate 95% confidence intervals per group. Large circles show group centroids. (**B**) Top 15 bacterial species by relative abundance. (**C**) Shannon index showing within-subject changes in alpha-diversity. Lines connect repeated measures from the same individual.

Five patients and seven controls had adequate samples for microbiome analysis, with metagenomic sequencing data averaging 4.5 million reads per sample. At baseline, patient samples showed greater within-group dispersion than controls and lower baseline diversity (Figure 3). Ten microbial pathways showed significant differences in relative abundance between patients and controls at baseline, including pathways involved in nucleotide, amino acid, and cell wall biosynthesis (Figure 3). All ten pathways had lower relative abundances in patient samples than in controls, suggesting a signature of reduced microbial fitness. Post-exercise, these relative abundances were no longer statistically significant. Individual patient responses were heterogeneous. Two patients exhibited increases in pathway abundance across all ten significant pathways to levels observed in controls, while others showed decreases. Species composition was altered in all samples post-exercise; however, there was not a uniform directional shift.

**Figure 4:**
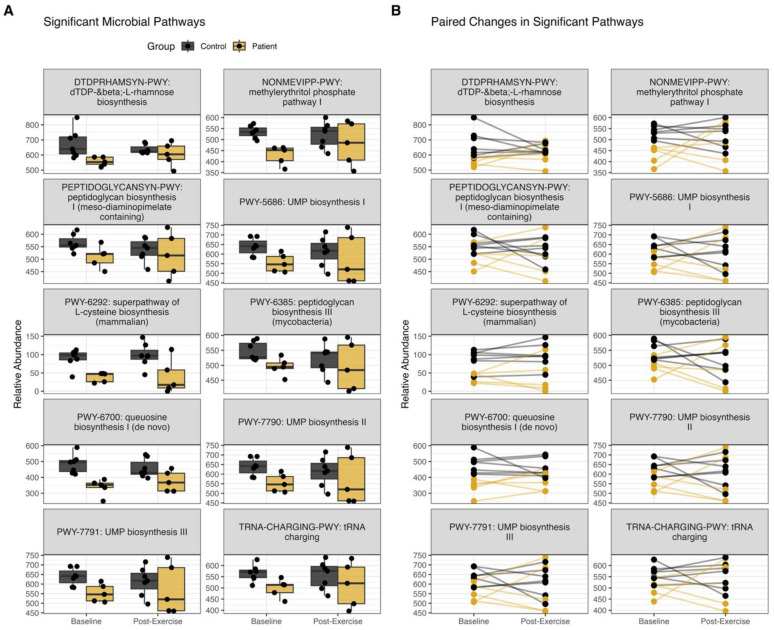
Significant pathways between controls and patients pre-exercise. (**A**) Boxplots of the relative abundance of each significantly differentially expressed pathway detected at baseline and post-exercise. (**B**) Paired changes in significant pathways, showing directionality of difference in detection of each pathway per subject.

Two patients had a marked increase in alpha diversity (Figure 3C) following resistance training, consistent with an increase in the relative abundance of pathways that synthesize amino acids, cell walls, and nucleotides, which may have been depleted during cancer treatment. While these patients likely experienced a healthy turnover of microbial communities, it is also possible that patients who decreased in diversity experienced some form of increased stability of gut microbes due to resistance training. Patient 3 (Figure 3B) showed an increase in *Fusicatenibacter saccharivorans*, which is known to produce short-chain fatty acids that strengthen intestinal barrier function [25]. Microbiome compositional differences and significant pathways between groups decreased post-exercise; however, dispersion among patient samples increased, highlighting differences in outcomes per patient (Figure 3 and Figure 4).

## 4. Discussion

In this community-based pilot study of pre-frail/frail cancer survivors and their healthy caregiver controls, a 10-week personalized resistance training program was found to be feasible, safe, and associated with meaningful improvements across multiple domains. Both cancer survivors and controls more than doubled their training volume, with no serious adverse events. Cancer survivors exhibited favorable changes in body composition, including reductions in body fat percentage and fat mass with preservation or gain of lean mass. At the transcriptomic level, cancer survivors demonstrated a pro-inflammatory, immunosenescent gene expression profile at baseline, characterized by elevated interferon signaling and reduced naïve T cell signatures, which was no longer significantly different from healthy controls after the intervention. Similarly, epigenetic differences between groups, particularly in genes related to naïve T cell biology, normalized after resistance training, and pathway analysis revealed exercise-associated hypomethylation of innate immune and Toll-like receptor genes in cancer survivors. Gut microbiome pathway differences present at baseline were also no longer statistically significant post-exercise. Taken together, these findings suggest that resistance training may exert anti-inflammatory and immune-restorative effects in cancer survivors beyond the well-established musculoskeletal benefits.

Cancer survivors can carry several risk factors for frailty, including advanced age, polypharmacy, lack of regular exercise, isolation, malnutrition, and unintentional weight loss [1,26]. The physiologic processes that underlie aging and result in frailty can all be worsened by cancer therapy [4]. Recent advances in cancer care are undeniably encouraging; however, the growing number of survivors may be lost in transition, and there is an ongoing need to improve the quality of life and recovery from therapy, as well as to reduce downstream risks from the treatment administered [27,28]. A physiological reason that exercise should be a cornerstone of cancer survivorship is that it is likely the most readily available therapy to mitigate the effects of aging [29,30,31,32]. The musculoskeletal benefits notwithstanding, we speculate that resistance training may reduce immunosenescence, particularly in recipients of allogeneic HCT, based on the resolution of epigenetic, transcriptomic, and microbiome differences observed after our exercise intervention. A recent mouse model demonstrated that exercise reversed age-induced changes in gene expression [29,32]. Our clinical trial results are consistent with these preclinical findings. Others have studied the influence of exercise on immunosenescence during aging [33,34,35,36]. Exercise has been shown to reduce proinflammatory markers in a large meta-analysis [37]. An important future direction will be to elucidate the underlying mechanisms driving these effects, which may ultimately inform the development of supportive therapies to preserve muscle mass and immune function in individuals unable to participate in resistance training due to comorbidities or other limitations. Additionally, while we found significant benefits of a 10-week program, the ideal duration of resistance training for optimal change remains unclear and warrants further study.

Our clinical trial results showed that the magnitude of strength gain was encouragingly similar between cancer survivors and controls, refuting the nihilistic notion that prior chemotherapy diminishes the ability to respond to resistance training stimuli. There are numerous ways to measure adaptation to strength training interventions, including maximum strength at a given number of repetitions (e.g., 1-repetition maximum), number of repetitions at a given weight, and training volume (the product of repetitions and weight moved). In this study, training volume was defined as the weight lifted multiplied by the number of repetitions performed in a single set. We chose volume as the measure of strength-training adaptation, rather than 1-repetition maximum, because it allowed a relatively untrained population to lift weights they were comfortable with within a repetition range they were accustomed to over the previous weeks of training. Measuring volume rather than a 1-repetition maximum also enhanced the safety of our study, reducing the risk of musculoskeletal injuries.

Additional features that enhanced feasibility included the intervention’s personalization and delivery in a commercial health club rather than a healthcare setting. Participants met at least once weekly for supervised group sessions but could train at locations closer to home during the week. Group sessions improved efficiency and reduced cost by allowing one trainer to supervise eight participants, while also fostering a sense of community and reducing isolation. Pairing cancer survivors with healthy caregivers may have further supported adherence and engagement, while also acknowledging the impact of cancer on caregivers and promoting healthier family-level behaviors. The pairing of cancer survivors with their caregivers, while intentionally designed to support adherence and provide a shared-exposure control group, may have introduced an additional emotional or motivational effect inherent to exercising as a dyad, which cannot be fully separated from the effects of the training intervention itself. As a single-arm, non-randomized before-and-after pilot study without a non-exercising cancer survivor control group, the design is susceptible to potential biases and confounding, including selection bias, the absence of blinding, maturation effects, and the possibility that observed changes may reflect natural recovery, regression to the mean, or other temporal factors rather than the exercise intervention alone. While all participants were at least 18 months post-treatment and the inclusion of healthy controls exposed to the same intervention and temporal conditions provides an internal comparator, we cannot fully exclude the contributions of natural physiologic changes over time, regression to the mean, or secondary behavioral effects of trial participation, such as changes in diet, alcohol or tobacco consumption, sleep, or sedentary behavior, to the observed outcomes. Fitbit-derived activity and sleep data did not differ significantly between groups and are provided in Supplementary Data, and nutritional counseling was standardized across all participants; however, not all lifestyle variables were formally monitored.

Multiple previous studies have demonstrated the benefits of exercise interventions for individuals recovering from cancer treatment, situating our findings within a well-established and growing body of evidence supporting exercise as a critical component of cancer survivorship care [38,39,40,41,42,43,44]. While we intended to recruit a broad range of patients, due to the timing of the study, we recruited mainly recipients of hematopoietic cell transplantation, who are exposed to high-dose chemotherapy and receive stem cells that reconstitute the immune system. This skewing of our population may have contributed to the results indicating resolution of inflammation and restoration of naïve T cell generation. Unfortunately, our study was terminated before the target sample size was accrued due to the emergence of SARS-CoV2 as a public health threat, particularly among immunocompromised individuals. Nonetheless, our pilot study results are encouraging: recipients of intensive chemotherapy and their caregivers can make significant gains in strength over a 10-week period, with the potential to restore cancer therapy-associated deficits in immune function through resistance training. With the recent finding that structured exercise improves survival among cancer survivors [13], future efforts should focus on further defining and confirming mechanisms of benefit, optimizing exercise programming to maximize physiologic benefit, and identifying methods to improve access to and long-term adherence to exercise programs.

## 5. Conclusions

In conclusion, our pilot study demonstrates that a community-based, personalized resistance training program is not only feasible for pre-frail/frail cancer survivors and their caregivers but also associated with meaningful improvements in strength, body composition, immune-related gene expression, DNA methylation, and microbiome profiles. Despite a small sample size, the consistency of gains across participants and the normalization of immune signatures suggest that resistance training may help reverse features of immune senescence induced by cancer therapy. These findings add to the growing evidence base supporting exercise as a cornerstone of survivorship care and highlight the potential of resistance training as a non-pharmacologic strategy to restore physiological resilience. Larger, longitudinal studies are now needed to validate these findings and explore sustainable, scalable models for delivering strength-based interventions to cancer survivors in diverse settings.

## Figures and Tables

**Table 1:** Baseline characteristics of cancer survivor participants and their healthy controls. Abbreviations: Allo = allogeneic, Auto = autologous, F = female, HCT = hematopoietic cell transplant, M = male.

Number	Group	Age	Sex	Treatment	# of Sessions	Included in Genomics Analysis
1	Cancer Survivor	62	F	Allo HCT	33	Yes
2	Control	59	M		34	Yes
3	Cancer Survivor	42	F	Allo HCT	29	Yes
4	Control	42	M		27	Yes
5	Cancer Survivor	57	M	Allo HCT	17	Yes
6	Control	27	F		23	Yes
7	Cancer Survivor	50	M	Allo HCT	27	Yes
8	Control	48	F		28	Yes
9	Cancer Survivor	48	M	Auto HCT	14	No
10	Control	48	F		12	No
11	Cancer Survivor	64	F	Breast Cancer	26	Yes
12	Control	59	F		30	Yes
13	Cancer Survivor	30	M	Allo HCT	8	No
14	Control	30	F		6	No
15	Cancer Survivor	35	M	Allo HCT	10	No
16	Control	28	F		9	No

**Table 2:** Top 10 cell types with lower gene expression in cancer survivors compared to controls prior to resistance training (CellMarker 2024).

Index	Name	Adjusted *p*-Value	Odds Ratio	Combined Score
1	Naive CD8+ T Cell Peripheral Blood Human	6.082 × 10^−14^	11.17	404.98
2	Naive CD4+ T Cell Peripheral Blood Human	6.082 × 10^−14^	27.06	980.17
3	Naive CD8+ T Cell Blood Human	4.098 × 10^−7^	28.29	568.55
4	CD8+ T Cell Lung Human	0.000004699	14.30	248.43
5	Central Memory CD8+ T Cell Blood Human	0.00004821	37.55	556.38
6	Naive CD8 T Cell Spleen Human	0.0002427	39.05	492.58
7	Naive CD8+ T Cell Liver Human	0.0002427	39.05	492.58
8	Central Memory CD8+ T Cell Liver Human	0.0002427	39.05	492.58
9	Central Memory CD8+ T Cell Spleen Human	0.0002427	39.05	492.58
10	Central Memory CD4+ T Cell Blood Human	0.0004256	31.24	373.20

**Table 3:** Top 10 pathways with high expression in cancer survivors before resistance training compared to post-exercise (WikiPathways 2024 Human).

Index	Name	Adjusted *p*-Value	Odds Ratio	Combined Score
1	Catabolism Of Skeletal Muscle In Cachexia WP5474	0.001396	10.55	132.61
2	miRNA Regulation Of Prostate Cancer Signaling WP3981	0.001396	12.29	147.53
3	Chromosomal And Microsatellite Instability In Colorectal Cancer WP4216	0.001396	6.72	79.08
4	Head And Neck Squamous Cell Carcinoma WP4674	0.005732	5.94	58.59
5	CAMKK2 Pathway WP4874	0.005732	4.70	46.01
6	Non Genomic Actions Of 1 25 Dihydroxyvitamin D3 WP4341	0.005732	5.67	54.14
7	Gastrin Signaling WP4659	0.005732	4.48	41.96
8	NAD Metabolism Sirtuins And Aging WP3630	0.005732	23.94	223.79
9	Nuclear Receptors In Lipid Metabolism And Toxicity WP299	0.005732	9.34	85.54
10	Wnt Signaling WP363	0.005732	7.37	67.45

**Table 4:** Pathway analysis of genes hypomethylated by exercise in cancer survivors (Reactome Pathways 2024).

Index	Name	Adjusted *p*-Value	Odds Ratio	Combined Score
1	Toll Like Receptor 3 (TLR3) Cascade	0.01996	8.47	75.89
2	MyD88-independent TLR4 Cascade	0.01996	8.14	71.30
3	TRIF (TICAM1)-mediated TLR4 Signaling	0.01996	8.14	71.30
4	TNF Receptor Superfamily (TNFSF) Members Mediating Non-Canonical NF-kB Pathway	0.02246	29.73	247.75
5	Toll-like Receptor Cascades	0.02246	5.94	48.28
6	MyD88 Cascade Initiated on Plasma Membrane	0.03263	7.53	54.09
7	Toll Like Receptor 10 (TLR10) Cascade	0.03263	7.53	54.09
8	Toll Like Receptor 5 (TLR5) Cascade	0.03263	7.53	54.09
9	Toll Like Receptor 4 (TLR4) Cascade	0.03263	5.95	42.66

**Table 5:** Top 10 cell marker pathways generated from the union of differentially methylated regions and differentially expressed genes in cancer survivors and controls before resistance training (CellMarker 2024).

Index	Name	Adjusted *p*-Value	Odds Ratio	Combined Score
1	Naive CD8+ T Cell Peripheral Blood Human	0.00003644	27.27	415.92
2	Naive CD4+ T Cell Peripheral Blood Human	0.0002244	48.27	614.98
3	Naive CD8+ T Cell Spleen Mouse	0.002290	255.68	2441.82
4	Quiescent T Stem Cell Skin Human	0.002290	191.75	1753.86
5	Naive T(Th0) Cell Colorectum Human	0.002290	191.75	1753.86
6	Naive CD8+ T Cell Bone Marrow Human	0.002290	153.39	1351.67
7	Naive CD4 T Cell Blood Human	0.002290	127.82	1089.78
8	Naive CD4 T Cell Liver Human	0.002290	127.82	1089.78
9	Naive CD4 T Cell Spleen Human	0.002290	127.82	1089.78
10	Naive CD4+ T Cell Blood Human	0.002290	127.82	1089.78

## Data Availability

The raw data supporting the conclusions of this article will be made available by the authors on reasonable request to newelll@ohsu.edu.

## Supplementary Materials

The following supporting information can be downloaded at https://www.mdpi.com/article/10.3390/cancers18111710/s1, Supplementary Data S1: Biometrics, ixercise, and Fitbit^®^ activity data; Supplementary Data S2: Differential gene expression results; Supplementary Data S3: Differential methylation results.
