# Supplementary material for: Attenuation of Immune Senescence Markers After Intensive Cancer Therapy Through Resistance Training: A Pilot Study
Source: Cancers (Basel). 2026 May 24;18(11):1710. doi: 10.3390/cancers18111710 (PMC13255878; doi:10.3390/cancers18111710)
Supplement: Supplementary file 1 [file cancers-18-01710-s001.zip › Supplemental Data S1- Biometric Exercise and FitBit.pdf]

Supplemental Data.

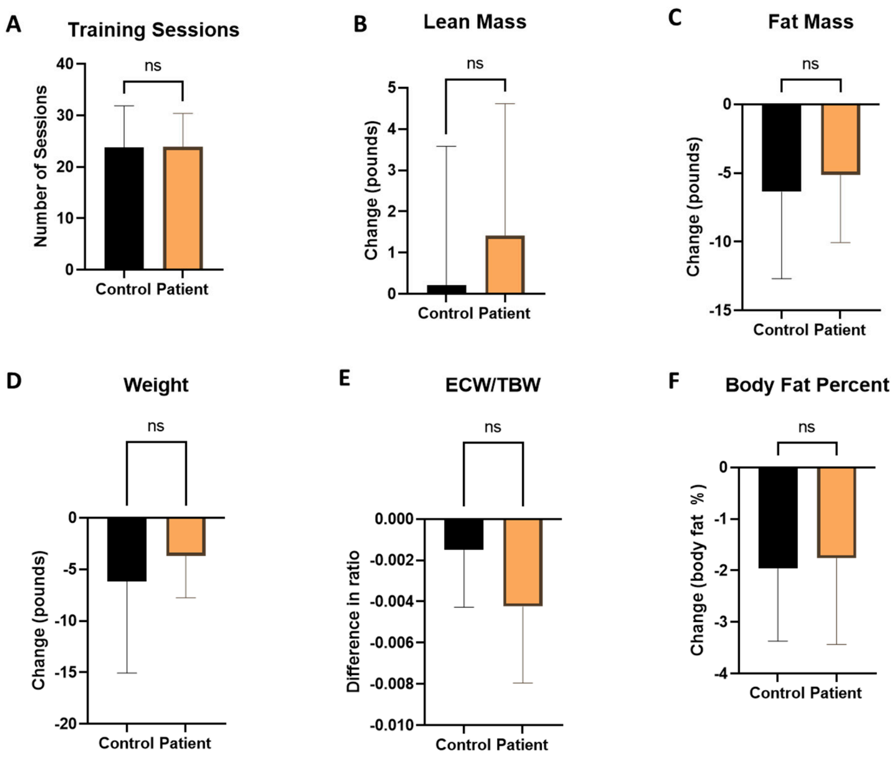

Supplemental Figure S1. Comparison of average +/- standard error of the mean (SEM) in (A) number of training sessions over 10 weeks, (B) lean body mass in pounds, (C) fat mass in pounds, (D) weight changed over 10 weeks in pounds, (E) change in the ratio of extracellular water (ECW) to total body water (TBW) as measured by bioelectrical impedance over 10 weeks, and (F) change in body fat percent over 10 weeks.

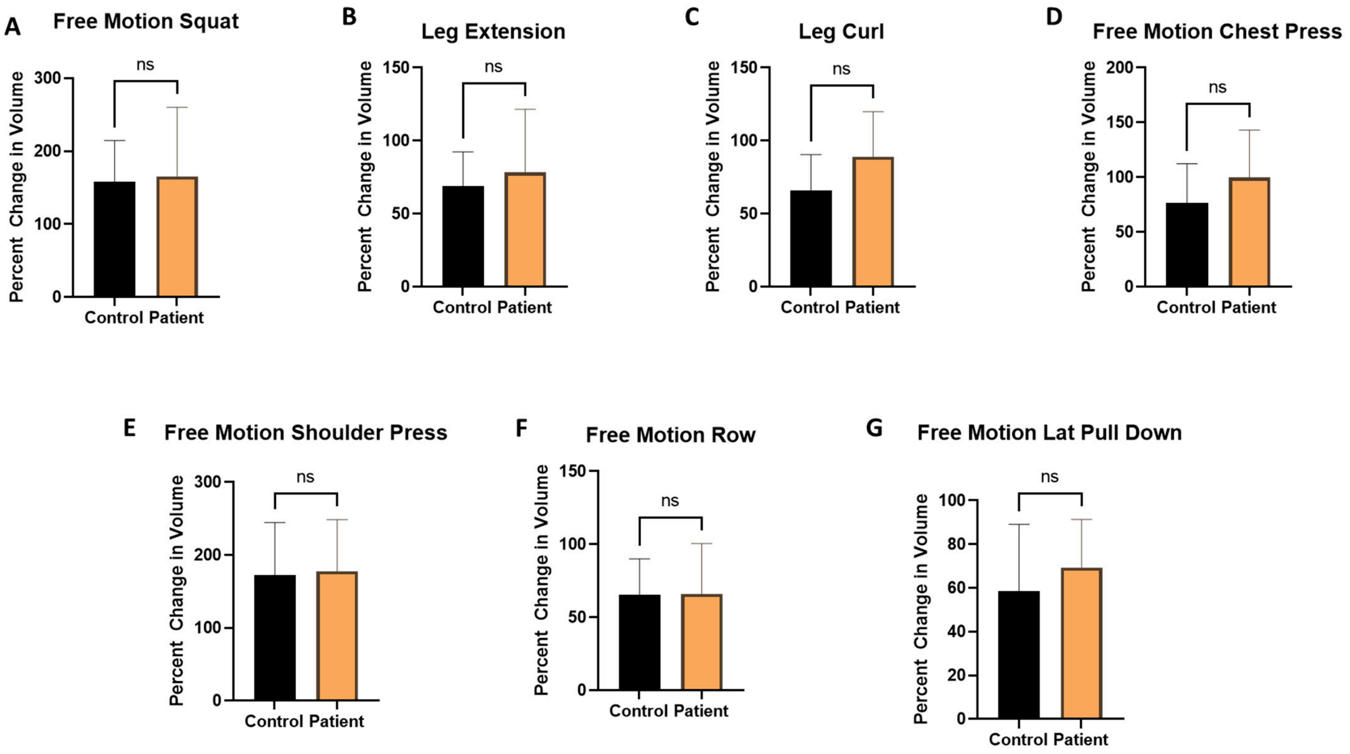

Supplemental Figure S2. Percent change in training volume (mean +/- SEM) from the baseline to final assessment as measured by 7 different resistance exercises (A-G).

Supplemental Table S1: Fitbit Accelerometer Data

Outcome	All subjects	Controls	Patients	P-value
Total number of steps				0.886
Mean (SD)	403516.2 (169026.4)	420560.2 (194017.2)	386472.2 (168064.8)	
Median (Range)	414618.0 (157615.0, 626603.0)	407569.0 (240500.0, 626603.0)	414618.0 (157615.0, 559038.0)	
Total number of steps (w/ 2 hour cutoff for wear time)				0.886
Mean (SD)	400369.6 (172527.0)	414436.0 (201716.1)	386303.2 (168035.6)	
Median (Range)	414314.5 (157546.0, 626597.0)	407472.5 (216202.0, 626597.0)	414314.5 (157546.0, 559038.0)	
Total number of steps (w/ 10 hour cutoff for wear time)				0.886
Mean (SD)	385216.1 (184480.8)	392312.0 (217642.0)	378120.2 (178629.1)	
Median (Range)	412115.5 (131622.0, 610786.0)	400859.0 (156744.0, 610786.0)	412115.5 (131622.0, 556628.0)	
Average number of steps per day				0.886
Mean (SD)	5953.1 (2028.3)	5923.4 (2732.6)	5982.8 (1459.4)	
Median (Range)	5839.7 (3387.3, 8825.4)	5740.4 (3387.3, 8825.4)	5839.7 (4378.2, 7873.8)	
Average number of steps per day (w/ 2 hour cutoff for wear time)				0.486
Mean (SD)	6963.3 (1452.2)	7386.6 (1814.2)	6540.0 (1073.2)	

Median (Range)	6534.1 (5525.0, 9214.7)	7394.0 (5543.6, 9214.7)	6380.6 (5525.0, 7873.8)	
Average number of steps per day (w/ 10 hour cutoff for wear time)				0.686
Mean (SD)	7453.1 (1386.2)	7976.3 (1600.7)	6929.9 (1091.4)	
Median (Range)	7004.4 (5525.0, 9396.7)	8062.9 (6382.7, 9396.7)	7004.4 (5525.0, 8185.7)	
Total distance in miles				0.886
Mean (SD)	281.3 (114.5)	290.4 (133.3)	272.2 (112.4)	
Median (Range)	291.6 (119.5, 432.4)	280.9 (167.4, 432.4)	291.6 (119.5, 386.0)	
Total distance in miles (w/ 2 hour cutoff for wear time)				0.886
Mean (SD)	279.1 (117.0)	286.1 (138.6)	272.0 (112.4)	
Median (Range)	291.4 (119.4, 432.4)	280.8 (150.5, 432.4)	291.4 (119.4, 386.0)	
Total distance in miles (w/ 10 hour cutoff for wear time)				0.886
Mean (SD)	268.3 (125.8)	270.8 (149.7)	265.9 (120.4)	
Median (Range)	289.8 (99.8, 421.4)	276.3 (109.1, 421.4)	289.8 (99.8, 384.2)	
Average distance in miles per day				0.886
Mean (SD)	4.2 (1.4)	4.1 (1.9)	4.2 (0.9)	
Median (Range)	4.1 (2.4, 6.1)	4.0 (2.4, 6.1)	4.1 (3.3, 5.4)	
Average distance in miles per day (w/ 2 hour cutoff for wear time)				0.686
Mean (SD)	4.9 (1.0)	5.1 (1.2)	4.7 (0.7)	

Median (Range)	4.7 (3.8, 6.4)	5.1 (3.9, 6.4)	4.7 (3.8, 5.4)	
Average distance in miles per day (w/ 10 hour cutoff for wear time)				0.686
Mean (SD)	5.2 (0.9)	5.5 (1.1)	4.9 (0.8)	
Median (Range)	5.2 (3.8, 6.5)	5.6 (4.4, 6.5)	5.2 (3.8, 5.7)	
Total very/moderately active minutes				>0.999
Mean (SD)	1098.9 (571.9)	1057.0 (845.0)	1140.8 (210.5)	
Median (Range)	1131.0 (149.0, 1969.0)	1055.0 (149.0, 1969.0)	1131.0 (948.0, 1353.0)	
Total very/moderately active minutes (w/ 2 hour cutoff for wear time)				>0.999
Mean (SD)	1098.9 (571.9)	1057.0 (845.0)	1140.8 (210.5)	
Median (Range)	1131.0 (149.0, 1969.0)	1055.0 (149.0, 1969.0)	1131.0 (948.0, 1353.0)	
Total very/moderately active minutes (w/ 10 hour cutoff for wear time)				>0.999
Mean (SD)	1059.2 (580.2)	1024.8 (864.8)	1093.8 (185.6)	
Median (Range)	1037.0 (58.0, 1931.0)	1055.0 (58.0, 1931.0)	1037.0 (948.0, 1353.0)	
Average very/moderately active minutes per day				0.686
Mean (SD)	17.7 (10.8)	14.9 (11.9)	20.5 (10.6)	
Median (Range)	16.4 (2.1, 35.8)	14.9 (2.1, 27.7)	16.4 (13.4, 35.8)	
Average very/moderately active minutes per day (w/ 2 hour cutoff for wear time)				0.686

Mean (SD)	20.7 (13.4)	17.5 (11.4)	23.9 (16.1)	
Median (Range)	17.2 (3.8, 47.8)	18.7 (3.8, 29.0)	17.2 (13.4, 47.8)	
Average very/moderately active minutes per day (w/ 10 hour cutoff for wear time)				0.886
Mean (SD)	22.5 (16.6)	18.2 (12.5)	26.8 (21.0)	
Median (Range)	17.9 (2.5, 58.0)	20.2 (2.5, 29.7)	17.9 (13.4, 58.0)	
Total estimated calories burned				0.886
Mean (SD)	153730.4 (48080.9)	164869.5 (53591.5)	142591.2 (46810.3)	
Median (Range)	134131.0 (94993.0, 223757.0)	161185.0 (113351.0, 223757.0)	134131.0 (94993.0, 207110.0)	
Average estimated calories burned per day				0.686
Mean (SD)	2347.2 (616.0)	2322.1 (754.8)	2372.3 (560.3)	
Median (Range)	2334.4 (1596.5, 3151.5)	2270.2 (1596.5, 3151.5)	2348.9 (1874.4, 2917.0)	
Total hours asleep				0.629
Number missing	1	1	0	
Mean (SD)	274.5 (175.7)	244.8 (195.7)	296.8 (186.3)	
Median (Range)	335.2 (20.3, 450.0)	335.2 (20.3, 379.0)	351.6 (33.8, 450.0)	
Average hours asleep per night				0.229
Number missing	1	1	0	
Mean (SD)	5.9 (2.0)	4.8 (3.0)	6.7 (0.1)	
Median (Range)	6.7 (1.4, 6.8)	6.4 (1.4, 6.7)	6.7 (6.5, 6.8)	

Total hours of light sleep				0.400
Number missing	1	1	0	
Mean (SD)	149.5 (103.2)	115.2 (102.2)	175.3 (110.8)	
Median (Range)	191.2 (0.0, 275.0)	150.6 (0.0, 195.1)	204.3 (17.6, 275.0)	
Average hours of light sleep per night				0.057
Number missing	1	1	0	
Mean (SD)	3.1 (1.4)	2.1 (1.8)	3.9 (0.3)	
Median (Range)	3.5 (0.0, 4.2)	3.0 (0.0, 3.3)	3.8 (3.5, 4.2)	
Total hours of deep sleep				0.857
Number missing	1	1	0	
Mean (SD)	45.3 (32.8)	42.2 (38.5)	47.7 (33.9)	
Median (Range)	51.2 (0.0, 83.8)	51.2 (0.0, 75.4)	51.1 (4.7, 83.8)	
Average hours of deep sleep per night				>0.999
Number missing	1	1	0	
Mean (SD)	0.9 (0.5)	0.8 (0.7)	1.0 (0.3)	
Median (Range)	0.9 (0.0, 1.4)	1.0 (0.0, 1.3)	0.9 (0.9, 1.4)	
Total hours of REM sleep				0.857
Number missing	1	1	0	
Mean (SD)	54.6 (37.1)	50.5 (44.2)	57.7 (37.7)	
Median (Range)	69.8 (0.0, 96.7)	69.8 (0.0, 81.8)	63.4 (7.4, 96.7)	

Average hours of REM sleep per night				0.629
Number missing	1	1	0	
Mean (SD)	1.2 (0.5)	0.9 (0.8)	1.3 (0.3)	
Median (Range)	1.4 (0.0, 1.6)	1.4 (0.0, 1.4)	1.4 (1.0, 1.6)	
Total hours of wear time				0.486
Mean (SD)	1092.2 (537.3)	962.0 (523.7)	1222.3 (595.0)	
Median (Range)	1357.0 (344.2, 1595.9)	996.1 (395.1, 1460.7)	1474.6 (344.2, 1595.9)	
Average hours of wear time per day				0.200
Mean (SD)	16.0 (6.8)	13.5 (7.4)	18.4 (6.1)	
Median (Range)	19.1 (5.6, 22.5)	14.0 (5.6, 20.6)	20.8 (9.6, 22.5)	
Average resting HR per day				0.686
Mean (SD)	69.4 (6.8)	68.2 (6.3)	70.5 (8.0)	
Median (Range)	66.2 (63.1, 82.0)	66.2 (63.1, 77.4)	67.8 (64.4, 82.0)	
Average peak HR per day				0.886
Mean (SD)	125.1 (13.9)	120.8 (6.3)	129.4 (19.1)	
Median (Range)	122.1 (112.5, 157.6)	122.1 (112.5, 126.7)	122.1 (115.9, 157.6)	
